# Advancing Rice Disease Detection in Farmland with an Enhanced YOLOv11 Algorithm

**DOI:** 10.3390/s25103056

**Published:** 2025-05-12

**Authors:** Hongxin Teng, Yudi Wang, Wentao Li, Tao Chen, Qinghua Liu

**Affiliations:** 1 College of Computer, Jiangsu University of Science and Technology, Zhenjiang 212003, China; 231210701120@stu.just.edu.cn; 2 College of Automation, Jiangsu University of Science and Technology, Zhenjiang 212003, China; 241710301101@stu.just.edu.cn (Y.W.); 241210301214@stu.just.edu.cn (W.L.); 241210301205@stu.just.edu.cn (T.C.)

**Keywords:** rice disease, object detection, intelligent agriculture, YOLOv11-RD, lightweight

## Abstract

Smart rice disease detection is a key part of intelligent agriculture. To address issues like low efficiency, poor accuracy, and high costs in traditional methods, this paper introduces an enhanced lightweight version of the YOLOv11-RD algorithm, enhancing multi-scale feature extraction through the integration of the enhanced LSKAC attention mechanism and the SPPF module. It also lowers computational complexity and enhances local feature capture through the C3k2-CFCGLU block. The C3k2-CSCBAM block in the neck region reduces the training overhead and boosts target learning in complex backgrounds. Additionally, a lightweight 320 × 320 LSDECD detection head improves small-object detection. Experiments on a rice disease dataset extracted from agricultural operation videos demonstrate that, compared to YOLOv11n, the algorithm improves mAP50 and mAP50-95 by 2.7% and 11.5%, respectively, while reducing the model parameters by 4.58 M and the computational load by 1.1 G. The algorithm offers significant advantages in lightweight design and real-time performance, outperforming other classical object detection algorithms and providing an optimal solution for real-time field diagnosis.

## 1. Introduction

Ensuring food security is pivotal for global peace and sustainability, with China contributing approximately a quarter of the world’s grain production [1]. As a staple food, rice is cultivated on 12% of the world’s arable land and sustains more than half of the global population [2,3,4]. However, the prevalence of rice diseases significantly impacts yield and quality, leading to substantial economic losses. Early and precise disease detection is essential for effective management [5], yet conventional manual inspection techniques are frequently imprecise, labor-intensive, and expensive. To address these challenges, computer-vision-based methods have been explored. Early approaches relied on traditional techniques such as background subtraction, template matching, and feature extraction. For instance, Qi et al. [6] integrated multi-scale feature fusion for small-target detection, while Bansal et al. [7] enhanced feature extraction using the Shi–Tomasi and SIFT algorithms. Despite their progress, these methods struggled with robustness in complex environments (e.g., varying growth stages, weed interference) and limited accuracy for small-scale diseases like Rice Blast [8].

The advent of deep learning has revolutionized rice disease detection. Single-stage models like the YOLO series [9,10,11] and SSD [12] excel in real-time processing, while two-stage frameworks such as Faster R-CNN [13,14,15] offer higher precision. Recent advancements focus on lightweight designs and attention mechanisms to tackle challenges in agricultural settings. For example, Xue et al. [16] improved YOLOv5 with global context modules, and Jia et al. [17] integrated MobileNetV3 into YOLOv7 for efficient feature extraction. However, existing models still face limitations in detecting subtle lesions under occlusion or dense planting, and their computational complexity hinders deployment on resource-constrained devices.

In 2024, the Ultralytics team launched YOLOv11, a lightweight model optimized for speed and adaptability. Building on this foundation, this study aims to develop a lightweight and efficient rice disease detection model, YOLOv11-RD, and deploy it in embedded agricultural equipment, thereby addressing issues such as untimely detection, missed detections, and false positives in rice disease identification, ultimately improving the yield of healthy rice. This study is closely related to intelligent agriculture and food security. By improving the accuracy and efficiency of disease detection, it can reduce yield losses caused by diseases and support precision agriculture decision-making. Moreover, the lightweight design of the model makes it suitable for deployment on resource-constrained field devices (such as drones or mobile terminals), promoting agricultural automation and sustainable development. Our key contributions include the following:

1. We introduced an enhanced attention module, SPPFLKC (an advanced version of the SPPF module with added Large-Separable-Kernel Attention and Convolution capabilities). When integrated with the backbone and neck, it significantly improves multi-scale feature extraction capabilities.
2. We added the C3k2-CFCGLU block (modified C3k2 block with CAFormer and CGLU), which consists of CAFormer (an efficient vision model combining convolution and self-attention) and the CGLU (an improved channel mixer), reducing computational complexity and channel redundancy while increasing the model’s proficiency in capturing fine-grained local features.
3. We designed the C3k2-CSCBAM (modified C3k2 block with CSCBAM) block based on the CSCBAM (Channel–Spatial Combined Attention Module), which reduces the training overhead and improves the network’s capability to learn target features in complex farmland environments.
4. We added the 320 × 320 scale LSDECD detection head, improved with detail-enhanced convolution, to enhance small-object detection capabilities while meeting lightweight requirements.

This study validates the performance of YOLOv11-RD on a diverse rice disease dataset, demonstrating its superiority in both accuracy and efficiency compared to state-of-the-art models. The remainder of this paper is organized as follows: Section 2 provides a detailed introduction to the YOLOv11-RD architecture; Section 3 presents the experiments and results; and Section 4 discusses the conclusions and future directions.

## 2. Model Architecture and Improvements

### 2.1. YOLOv11 Architecture

**Figure 1:**
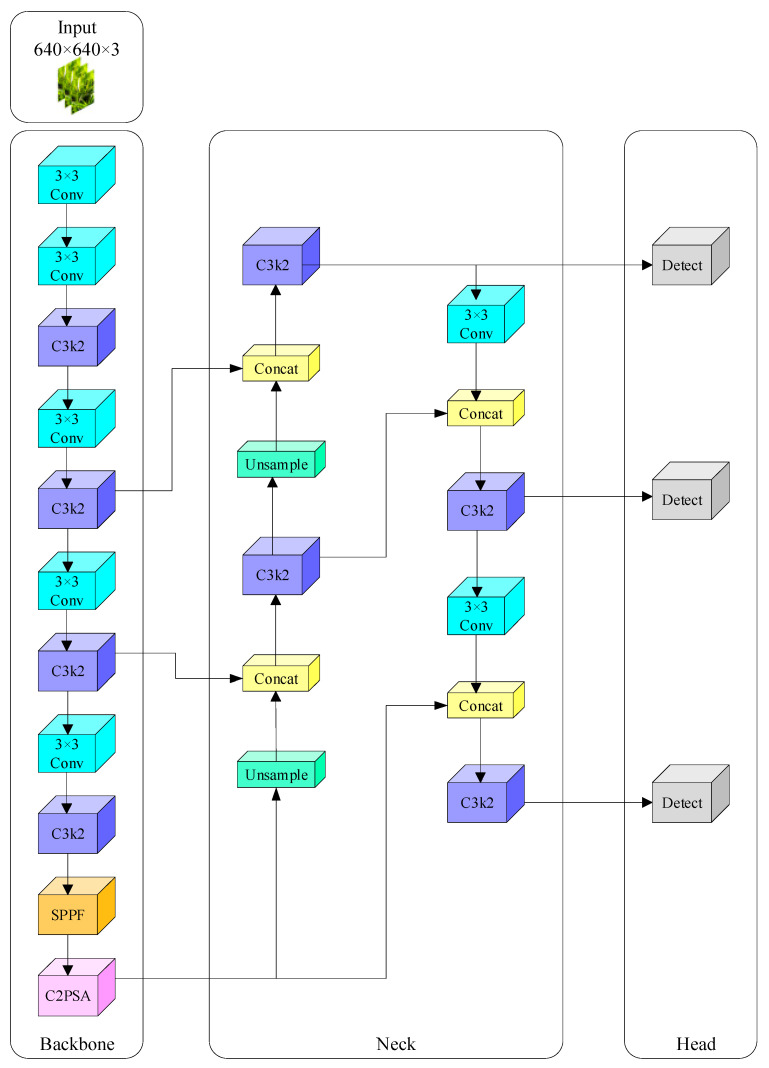
YOLOv11 architecture: backbone, neck, and detection head design.

YOLOv11 [18] builds on the capabilities of its predecessors, with YOLOv11n specifically optimized for environments with limited resources. The key distinction of YOLOv11n lies in its parameter count, computational load, and overall size, making it suitable for resource-constrained devices. Figure 1 illustrates the architecture of YOLOv11n. Compared to YOLOv8, YOLOv11 represents a significant advancement by replacing YOLOv8’s C2f structure [19] with the C3k2 (Cross Stage Partial Bottleneck with 3 Conv layers and Key-point Detection) structure in YOLOv11n. This new structure enhances feature capture comprehensively, boosting the convolutional neural network’s feature extraction capability while improving the computational efficiency and further reducing the model size. For YOLOv11n, the C3k2 structure dynamically adjusts the number of channels to better accommodate inputs of various sizes, ensuring adaptability and performance in diverse scenarios.

The key improvement in YOLOv11 is the replacement of the C2f structure with the C3k2 structure, which enhances feature extraction capabilities and improves computational efficiency.

### 2.2. YOLOv11-RD Architecture

**Figure 2:**
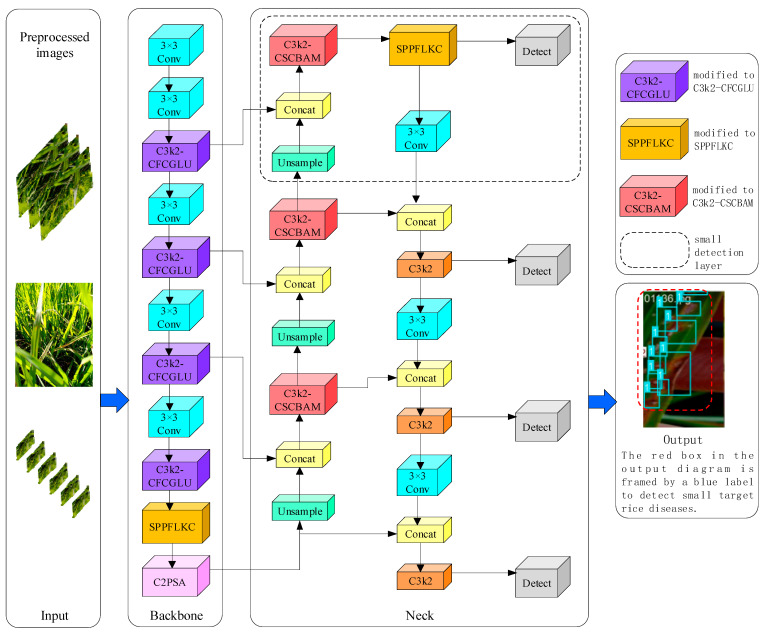
YOLOv11-RD improved architecture: integration of SPPFLKC module, C3k2-CFCGLU, C3k2-CSCBAM, and lightweight detection head.

For rice disease detection tasks, agricultural field equipment (such as portable detection devices) typically requires models to be lightweight, low-latency, and highly accurate. Such a model can swiftly and precisely identify rice diseases in complex farmland environments, ensuring the timely implementation of disease management measures to protect the healthy growth and yield of rice. The YOLOv11-RD architecture introduced in this paper, as shown in Figure 2, includes four parts: the input, backbone, neck, and output.

YOLOv11-RD optimizes the original YOLOv11 architecture, enhancing small-object detection performance while achieving model lightweighting without compromising detection capabilities. The key improvements introduced in this paper are listed below:

In the backbone network, an enhanced LSKAC (Large-Separable-Kernel Attention with Convolution) module is integrated with SPPF (Spatial Pyramid Pooling Fast) to form the new SPPFLKC module, replacing the original SPPF. This not only enhances feature representation but also boosts the feature extraction proficiency of the rice disease detection model in complex farmland settings.

Similarly, in the backbone, C3k2 is substituted with C3k2-CFCGLU, which strengthens the model’s capability to capture local details, reduces the computational overhead and storage requirements, and balances performance with model size.

In the neck section, the upsampling C3k2 is replaced by the C3k2-CSCBAM, based on the CSCBAM. By maintaining high-performance feature extraction, this approach simultaneously results in a marked decrease in model parameter count, thereby streamlining the overall architecture.

Similarly, in the neck (illustrated by the dashed box in Figure 2), an innovative lightweight LSDECD detection layer is incorporated. This layer not only utilizes the C3k2-CSCBAM but also embeds the SPPFLKC attention mechanism during the downsampling process, significantly elevating the detection accuracy for minute objects.

### 2.3. SPPFLKC Attention Mechanism

#### 2.3.1. LSKAC

The Large-Separable-Kernel Attention (LSKA) [20], proposed in 2023, stands as a cutting-edge attention mechanism for visual tasks. Evolving from the original LKA [21] mechanism, LSKA demonstrates exceptional capability in handling long-range dependencies within images, significantly enhancing spatial and channel adaptability in deep networks. LSKA significantly cuts down on computational expenses, leading to a marked improvement in the model’s operational efficiency.

Expanding on the foundational LSKA architecture, researchers have innovatively dissected and refined the core convolution operations to transcend the inherent limitations of self-attention mechanisms and large-kernel convolutions. This enhancement notably boosts the model’s capability to capture long-range spatial dependencies, resulting in an enhanced LSKAC module. This module combines the global information perception benefits of self-attention with the broad-range sensing capabilities of large-kernel convolutions. By streamlining computations and reducing parameter counts, it significantly boosts the model’s ability to recognize long-range dependencies, reduces resource usage, and boosts processing efficiency, ensuring good performance under resource constraints.

**Figure 3:**
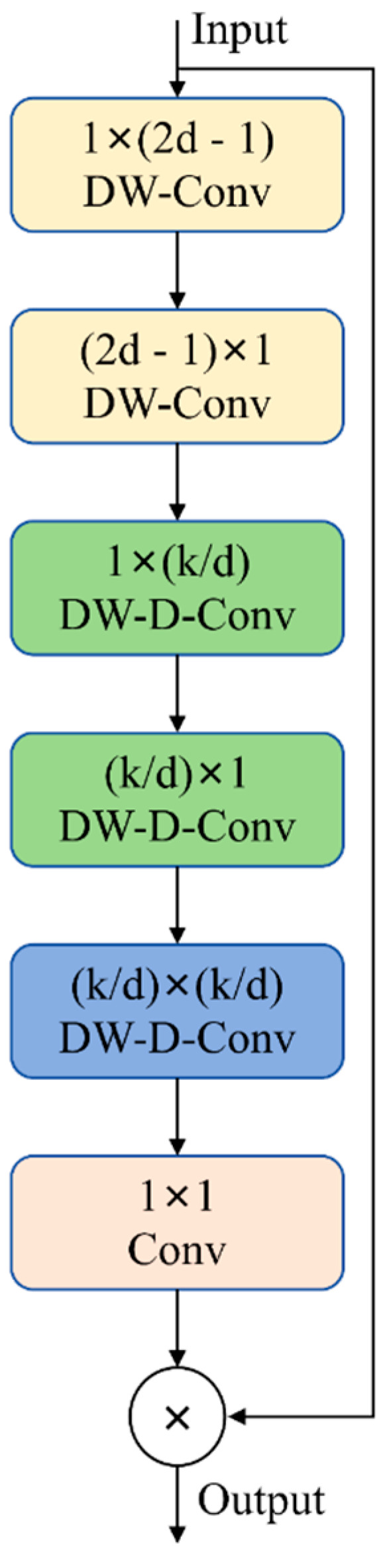
Structure of LSKAC.

The LSKAC module identifies long-range dependencies through the efficient decomposition of large-kernel convolutions, requiring minimal computation and parameters. The large-kernel convolution is decomposed into three parts: spatial local convolution (depth-wise convolution), termed DW-Conv; spatial remote convolution (depth-wise dilated convolution), labeled DW-D-Conv; and channel-wise 1 × 1 convolution, abbreviated as Conv. Figure 3 shows the LSKAC network structure. First, a 1 × (2*d* − 1) depth-wise separable convolution captures vertical features and reduces parameters. Then, a (2*d* − 1) × 1 depth convolution extracts horizontal features, completing the previous step. Next, vertical sparse convolutions are performed using a depth-wise separable sparse convolution with a 1 × (*k*/*d*) kernel, broadening the receptive field to detect distant features. Similarly, a (*k*/*d*) × 1 depth-wise separable sparse convolution expands the receptive field horizontally. In the 2D direction, a (*k*/*d*) × (*k*/*d*) depth-wise-expanded convolution further integrates these features. Finally, a 1 × 1 convolution merges the extracted features, reduces the number of channels, and performs element-wise multiplication with the original input feature map to generate the output.

Considering an input feature map *F* ∈ *R^C^^×H^^×W^*, where *C* signifies the number of channels, and *H* and *W* denote the dimensions of the feature map, the advanced LSKAC module is achieved by transforming the traditional 2D depth-wise convolution kernel into two sequentially applied 1D separable convolution kernels. By leveraging Equations (1) through (4) along with a dilation factor *d*, the LSKAC output is determined. The input feature map *F^C^* is convolved with two cascading 1D depth-wise separable convolution kernels W of sizes 1 × (2*d* − 1) and (2*d* − 1) × 1. Each channel *C* within *F* is aggregated with its corresponding channel in *W*. Consequently, the output ${\bar{Z}}^{C}$, which encapsulates the local spatial attributes, is formulated as follows:

(1) $${\bar{Z}}^{C}=\sum_{H,W}W_{(2d-1)\times 1}^{C}*\left(\sum_{H,W}W_{1\times (2d-1)}^{C}*F^{C}\right)$$

The output ${\bar{Z}}^{C}$, generated via Equation (1), is subjected to convolution with the large-kernel decomposition of the depth-wise-expanded convolution *W*, utilizing a kernel size of [*k*/*d*][*k*/*d*][*k*/*d*][*k*/*d*]. This convolutional operation serves to effectively counteract the lattice effect that arises from the depth-wise-expanded convolution. The resulting ${\bar{Z}}^{C}$ is then calculated in the following manner:

(2) $$Z^{C}=\sum_{H,W}W_{\left\lfloor \frac{k}{d}\right\rfloor \times \left\lfloor \frac{k}{d}\right\rfloor }^{C}*{\bar{Z}}^{C}$$

The output $Z^{C}$ from Equation (2) is convoluted with a 1 × 1 convolution kernel *W*, resulting in the attention map *A^C^*, which is computed as follows:

(3) $$A^{C}=W_{1\times 1}*Z^{C}$$

Ultimately, the attention map *A^C^* obtained from Equation (3) is element-wise-multiplied with the input feature map *F^C^*, yielding the output ${\bar{F}}^{C}$, as per Equation (4):

(4) $${\bar{F}}^{C}=A^{C}⊙F^{C}$$

Here, * and ⊙ represent convolution and the Hadamard product (element-wise multiplication), respectively. This decomposition reduces the feature extraction costs to those of depth-wise convolutions and larger kernels, thereby curbing the increase in the computational cost.

Assuming the input and output feature map sizes for LSKA and LSKAC are identical, Equations (5)–(8) provide the formulas to compute the floating-point operations and parameters for LSKAC and LSKA, with *k* representing the kernel size and *d* denoting the dilation rate. Comparing Equations (5) with (7) and Equations (6) with (8), it is evident that the proposed LSKAC retains an additional (2*d −* 1)/2 effective parameters compared to the initial LSKA. Consequently, LSKAC achieves higher accuracy in feature extraction:

(5) $$Param=(2d-1)\times C\times 2+{\left\lfloor \frac{k}{d}\right\rfloor }^{2}\times C+C\times C$$

(6) $$\mathrm{FLOPs}=\left((2d-1)\times C\times 2+{\left\lfloor \frac{k}{d}\right\rfloor }^{2}\times C+C\times C\right)\times H\times W$$

(7) $$Param={\left(2d-1\right)}^{2}\times C+{\left\lfloor \frac{k}{d}\right\rfloor }^{2}\times C+C\times C$$

(8) $$\mathrm{FLOPs}=\left({\left(2d-1\right)}^{2}\times C+{\left\lfloor \frac{k}{d}\right\rfloor }^{2}\times C+C\times C\right)\times H\times W$$

In summary, LSKAC capitalizes on the strengths of large-kernel convolutions and self-attention to improve the model’s flexibility and adaptability across spatial and channel dimensions.

#### 2.3.2. SPPFLKC

**Figure 4:**
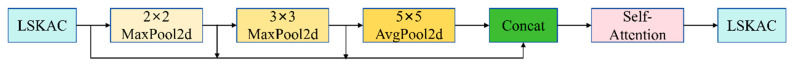
Structure of SPPFLKC.

Figure 4 below depicts the SPPFLKC attention mechanism module, reconstructed with LSKAC and comprising LSKAC, MaxPool2d, AvgPool2d, self-attention, and Concat layers.

The SPPFLKC attention mechanism substitutes the convolutional layers in the conventional SPPF with an integrated LSKAC module, enabling automatic feature selection. This approach integrates local context information, large receptive fields, and dynamic feature variations to selectively highlight important features and remove irrelevant noise based on input data. This significantly enhances feature representation quality. The SPPFLKC module, by integrating LSKAC and other components, significantly enhances the model’s feature extraction capabilities in complex environments. Specifically, it expands the receptive field and improves the selectivity of important features, making it highly suitable for small-target detection and various complex scenarios. For example, when detecting subtle diseases like Rice Blast, SPPFLKC effectively captures critical features, reducing false positives. As a result, incorporating the optimized SPPFLKC attention mechanism with YOLOv11 for small-object detection is deemed both practical and effective.

### 2.4. C3k2-CFCGLU

SCConv (Split Convolution) [22] was proposed in 2023 to reduce computational demands caused by redundant feature computation during visual processing. However, the original SCConv experiences a decline in accuracy and fails to adequately reduce superfluous information. To address these issues, the C3k2-CFCGLU module incorporates concepts from depth-wise separable convolution [23] and gated channel attention [24] into the original SCConv, creating CAFormer and the CGLU to further enhance the model.

**Figure 5:**
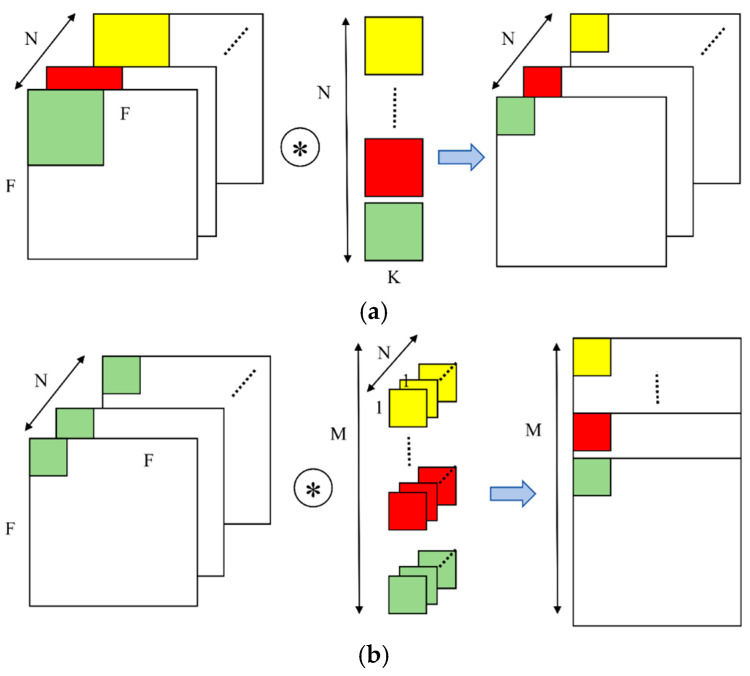
Depth-wise separable convolution.

**Figure 6:**
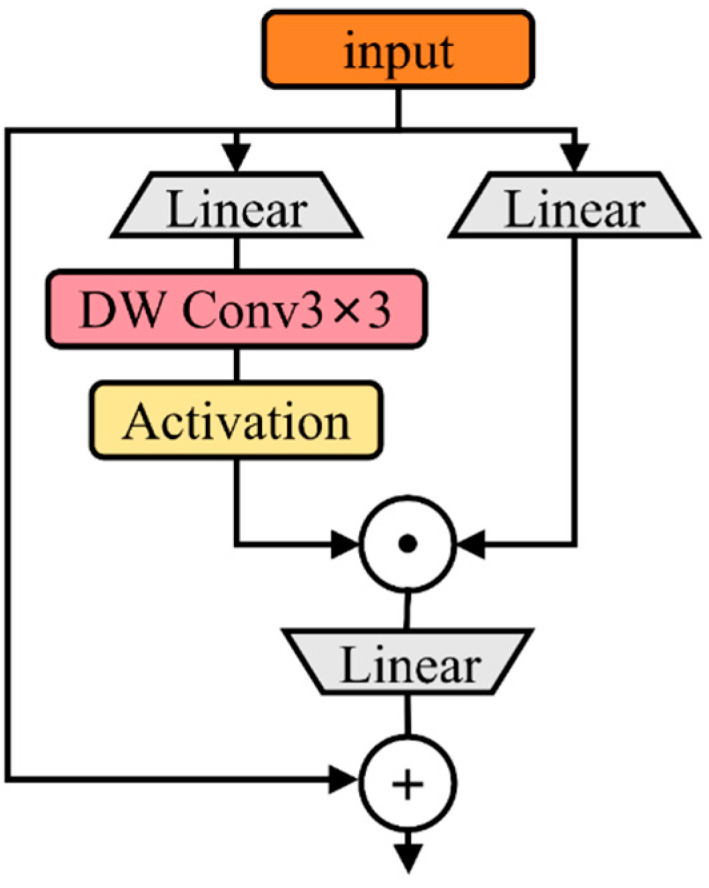
Gated channel attention.

Depth-wise separable convolution splits the standard n × n convolution into two distinct operations, as illustrated in Figure 5. Part (a) shows depth-wise convolution, which applies convolution independently to each input channel, while part (b) depicts point-wise convolution using a 1 × 1 kernel to project the depth-wise convolution results into a new feature space. This method markedly lowers the computational demands as well as the parameter quantity, enabling the more efficient extraction of comprehensive target features. Figure 6 illustrates the concept of gated channel attention, where the mechanism uses a gating signal to dynamically identify important features and suppress irrelevant noise, thereby enhancing the quality of feature representations.

**Figure 7:**
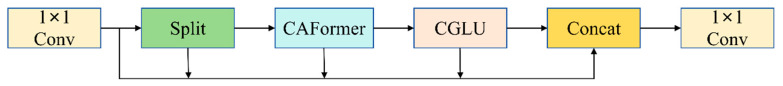
C3k2-CFCGLU structure.

In the C3k2-CFCGLU module, depth-wise separable convolution is first applied to extract local features, followed by gated channel attention to dynamically select important features, enhancing the precise extraction of useful information. Figure 7 shows that the C3k2-CFCGLU module sequentially swaps the two initial bottlenecks for CAFormer and the CGLU. This component decreases the model parameters and processing expenses while also efficiently minimizing feature redundancy through enhanced feature representations.

#### 2.4.1. CAFormer

**Figure 8:**
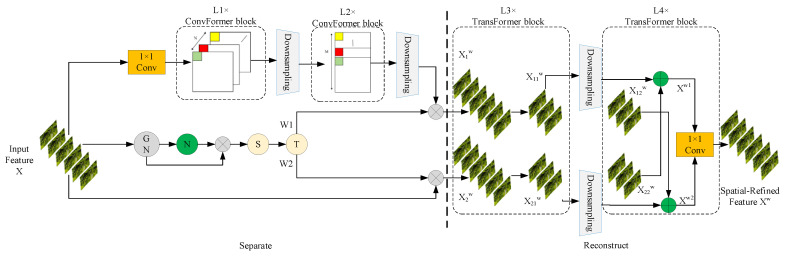
Architecture of CAFormer.

Figure 8 illustrates CAFormer (a neural network architecture that integrates convolution and self-attention mechanisms), which employs a dual-branch setup to improve feature extraction by combining depth-wise separable convolutions with self-attention mechanisms. The entire process is partitioned into two principal phases: feature separation and feature reconstruction.

In CAFormer, the input feature map is denoted as *X* ∈ *R^C×H×W^*, where *N* is the batch dimension, *C* is the channel dimension, and *H* and *W* are the spatial height and width dimensions, respectively. The complete architecture is split into two components: the separation part and the reconstruction part. The separation part incorporates depth-wise separable convolutions. In the sub-branches, convolution layers use learned offsets to adjust the sampling points of the main branch’s convolution layers. The input feature map *X* first undergoes a 1 × 1 convolution layer for dimensionality reduction; then, depth-wise convolutions are applied for initial processing, producing the *L*1 feature map. After downsampling, point-wise convolutions further process the feature map, producing the *L*2 feature map and performing additional downsampling. In the primary branch, the learnable parameter *γ* ∈ *R^C^* from GN indicates pixel variation in each batch and channel space. Following N layers, normalization generates batch- and channel-specific weights *W_γ_* ∈ *R^C^*, which are subsequently multiplied with the feature map processed by the GN layer. Next, a Sigmoid function generates weights *W_γ_* in the range of (0, 1), controlled by a thresholding layer *t*, resulting in two types of weights: informative weights *W*_1_ (exceeding the threshold) and non-informative weights *W*_2_ (below the threshold). This separates the information and forms two branches.

During reconstruction, the feature map from depth-wise separable interpolation is multiplied by W_1_, and the input feature map *X* is multiplied by *W*_2_, producing two weighted feature maps: *X*_1_*^W^*, which contains rich and expressive spatial information, and *X*_2_*^W^*, which encompasses insignificant and repetitive data. Then, a cross-reconstruction operation blends *X*_1_*^W^* and *X*_2_*^W^*, enhancing feature interaction. Lastly, the cross-reconstructed feature maps *X*_1_*^W^* and *X*_2_*^W^* are concatenated, yielding the spatially refined feature map *X^W^*. Equations (9)–(13) outline the reconstruction procedure:

(9) $$X_{1}^{W}=W_{1}⊗X$$

(10) $$X_{2}^{W}=W_{2}⊗X$$

(11) $$X_{11}^{W}⊕X_{22}^{W}=X^{W1}$$

(12) $$X_{21}^{W}⊕X_{12}^{W}=X^{W2}$$

(13) $$X^{W1}\cup X^{W2}=X^{W}$$

The feature map processed by CAFormer efficiently distinguishes key features from secondary components and enhances feature expressiveness through multiple reconstruction steps, reducing spatial redundancy. However, even after spatial refinement, some redundancy persists in the channel dimension of the feature map.

#### 2.4.2. CGLU

**Figure 9:**
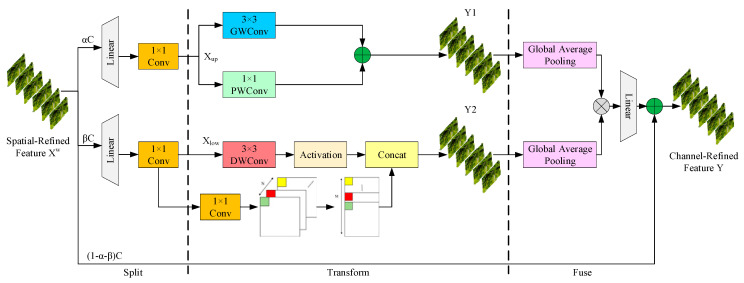
Architecture of CGLU.

Figure 9 shows the three-branch architecture of the CGLU, combining convolution and gating mechanisms. Features pass through three stages. During segmentation, the CAFormer output (represented as *X* ∈ *R^ᴄ × H × W^*) is fed into the CGLU to obtain diverse and typical features. Initially, the channels of X are split into three segments, with parameters *α* and *β* (0 ≤ *α*, *β* ≤ 1) managing the channel distribution ratio, balancing computational cost and efficiency. Next, a linear layer followed by a 1 × 1 convolution is applied to reduce the channel dimensions of the feature map, boosting computational efficiency. After segmentation and compression, the feature *X^W^* is split into the upper branch (*Xup*) and the lower branch (*Xlow*).

During the transformation phase, *Xup* serves as a “rich feature extractor”, using parallel group-wise convolution (GWConv) and point-wise convolution (PWConv) to obtain feature details while minimizing computational load. GWConv restricts information flow between channel groups to lower the computational burden, while PWConv remedies any information loss. Aggregated from the generated feature outputs, the feature map *Y*1 is formed. In the lower transformation stage, *Xlow* is processed by a 3 × 3 depth-wise separable convolution layer, reducing the computational burden and parameter count while improving feature characterization. A nonlinear activation function boosts expressiveness, and depth-wise separable interpolation with concatenation extracts subtle details from *Xlow*, augmenting information from the upper branch, culminating in the output *Y*2.

After transformation, Y1 and Y2 undergo global pooling to aggregate spatial features, followed by fusion and refinement via a linear layer for enhanced representation. These refined features are combined with selective elements from a third branch, resulting in the channel-refined feature Y.

At its core, the CGLU uses a tripartite approach to minimize channel redundancy. It optimizes resource allocation via efficient segmentation and compression, enriches feature extraction with lightweight convolutions, and enhances efficiency through cost-effective feature reuse. This strategy reduces redundancy while significantly boosting the network’s feature representation ability.

Overall, the C3k2-CFCGLU block combines depth-wise separable convolution with gated channel attention to achieve efficient feature extraction and reduce redundancy. This not only improves model accuracy but also significantly lowers the computational overhead, making the model more suitable for deployment on resource-constrained devices while maintaining real-time detection capability.

### 2.5. C3k2-CSCBAM

**Figure 10:**
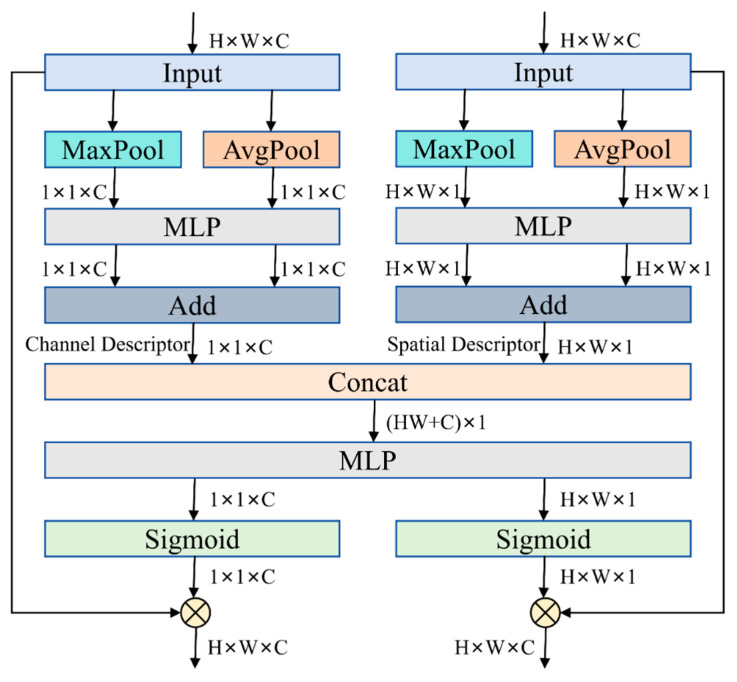
Architecture of CSCBAM.

To address accuracy degradation and insufficient feature fusion in the CBAM, an innovative joint fusion approach [25] was proposed, seamlessly integrating channel and spatial attention to enhance their interaction. This method introduces the CSCBAM, optimizing channel representation using spatial attention based on advanced fusion techniques. Figure 10 shows the CSCBAM architecture, comprising three key stages: compression, interaction, and separation.

During compression, channel attention and spatial attention operations show notable similarities, both aiming to streamline data and extract essential features. Both utilize max pooling (Max Pool) and average pooling (Avg Pool) to derive information from the input feature map. For channel attention, these two pooling operations generate two 1 × 1 × *C* channel descriptors. For spatial attention, they produce two *H* × *W* × 1 spatial descriptors. Next, these descriptors are processed by a multi-layer perceptron (MLP) and then fused using an addition operation (Add) to generate the final channel descriptor and spatial descriptor.

In the interaction stage, the previously obtained channel descriptor (1 × 1 × *C*) and spatial descriptor (*H* × *W* × 1) are concatenated (Concat) to form a comprehensive feature representation with a size of (*HW* + *C*) × 1. Then, another multi-layer perceptron (MLP) is used to model this combined feature, capturing the correlation between global spatial features (i.e., the channel descriptor) and global channel features (i.e., the spatial descriptor).

In the separation stage, the fully interacted global spatial and channel features are split back into their original shapes: 1 × 1 × *C* and *H* × *W* × 1. These features are scaled to the 0–1 interval with a Sigmoid function, generating the attention weights. Ultimately, these weights are multiplied with the original input feature map to generate the final output after weighted adjustment.

Briefly, the CSCBAM ensures excellent detection performance while markedly lowering the parameter count with an efficient fusion mechanism. By employing a three-stage structure—compression, interaction, and separation—this module seamlessly integrates channel and spatial attention, enabling more comprehensive feature extraction. This joint modeling approach sharpens the model’s emphasis on essential features, thereby boosting overall performance.

**Figure 11:**
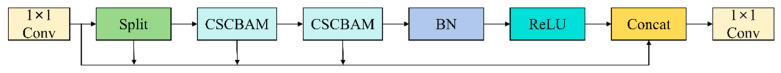
Structure of C3k2-CSCBAM.

Figure 11 displays the design of the C3k2-CSCBAM, in which the bottleneck layer is replaced by the CSCBAM. To maintain optimization diversity and training stability, a BN layer and a ReLU layer are inserted prior to the fusion process. Although BN and ReLU layers enhance model performance, the excessive use of these layers can add significant computational load during training and may result in overly large model weights. Therefore, to improve computational efficiency, the C3k2-CSCBAM utilizes a sole BN layer and a ReLU layer, markedly decreasing training costs while sustaining model stability and optimization efficiency.

The C3k2-CSCBAM block integrates channel and spatial attention mechanisms to enhance feature fusion in complex backgrounds. By reducing the number of parameters, this module improves the model’s performance in detecting small diseases, especially under dense planting conditions, where it can better identify and distinguish disease features, adapting to various complex scenarios.

### 2.6. Small Detection Layer

This research centers on detecting small objects in paddy fields, such as diseased rice plants and weeds. The YOLOv11 model generates feature maps at 20 × 20, 40 × 40, and 80 × 80 resolutions, which are not effective for small-object detection. To tackle this issue, a refined version of YOLOv11 was developed by adding a 320 × 320 layer tailored for detecting small objects (shown within the dash-bordered area in Figure 2). The enhanced YOLOv11-RD obtains features from the backbone network’s sixth layer and merges these with contextual details obtained via the SPPFLKC attention mechanism using a Concat operation. This results in a dedicated fourth detection head tailored for small-object detection. This detection head enhances feature extraction through the SPPFLKC attention mechanism, reducing false positives and negatives, thereby significantly improving the detection accuracy of small targets in the field. Additionally, this design ensures efficient operation on resource-constrained devices, supporting real-time detection requirements.

## 3. Results and Discussion

### 3.1. Dataset Preparation

This study aims to improve rice disease detection accuracy by addressing the limitations in existing datasets, such as insufficient disease diversity, suboptimal image quality, and inadequate annotations. In collaboration with agricultural research institutions, 5000 raw images covering Brown Spot, Rice Blast, and Bacterial Blight (see distribution in Table 1) were extracted from farming videos. Through rigorous selection criteria, 3000 high-quality images were retained: excluding 15% of low-quality samples (blurred, overexposed, or occluded) while ensuring a resolution of ≥1280 × 720 and clear feature visibility; covering diverse disease stages (e.g., Brown Spot from punctate to necrotic lesions), environmental conditions (natural/artificial lighting, sunny/rainy/foggy), and plant parts (leaves, stems, panicles); and removing 5% of ambiguous annotations (bounding box IoU < 0.8) via cross-validation by three experts. Stratified sampling balanced the disease classes (±5% error), and 10% challenging samples (e.g., tiny lesions, inter-class similarities) were included to enhance robustness. The meticulously annotated dataset provides a representative and practical foundation for training detection models in complex field scenarios.

**Figure 12:**
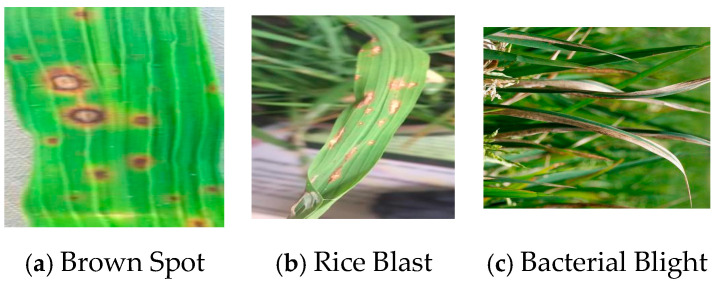
Three typical rice disease images.

To prevent overfitting, data augmentation techniques like random flipping, contrast adjustment, and Gaussian blur were used during training, expanding the dataset to 4000 images. All images were annotated with LabelImg, and the class distribution was balanced at 4:3:3. The category distribution is detailed in Table 2. Figure 12 shows images of three typical rice diseases after data augmentation, with their lesions displaying clear differences.

The dataset of 4000 images is split into training (2800), validation (800), and test sets (400) at a 7:2:1 ratio for balanced distribution. To enhance robustness and efficiency, all images were resized to 640 × 640 pixels and preprocessed with normalization for consistency.

This dataset has been carefully optimized to improve clarity and contour definition. These improvements enable the more reliable evaluation of rice disease detection algorithms in complex scenarios, such as heavy occlusions or poor lighting, significantly increasing the dataset’s complexity.

### 3.2. Experimental Environment and Parameter Setup

The experimental setup runs on Windows 10, using an AMD EPYC 9754 processor (128 cores), 16GB RAM, and an Nvidia GeForce RTX 4090D GPU (24GB GDDR6X). With PyTorch 1.11.0, Python 3.8, and CUDA 11.3 for acceleration, this configuration ensures high performance. Table 3 lists the relevant parameter settings, providing insights into the experimental design.

### 3.3. Evaluation Metrics

To comprehensively evaluate model performance, we selected precision (P), recall (R), and mean average precision (mAP) to measure detection accuracy, with higher values indicating better performance. We also considered the computational complexity (GFLOPs) and the number of parameters to assess the model’s lightweight features, where lower values signify higher efficiency and reduced hardware requirements, which is crucial for deployment on embedded devices.

### 3.4. Evaluation Results

To assess the efficacy of the SPPFLKC attention mechanism, this experiment integrates C3k2-CFCGLU, C3k2-CSCBAM, and a small-object detection head. Ablation and comparative experiments are conducted to analyze how these enhancements impact the performance of the YOLOv11n model.

#### 3.4.1. SPPFLKC Validity Analysis

This paper compares the SPPFLKC attention mechanism with other prominent mechanisms under consistent conditions (the same configuration and dataset), such as SE [26], ELA [27], ECA [28], EMA [29], LSKA [30], and SimAM [31].

As shown in Table 4, SPPFLKC outperforms mainstream attention mechanisms such as ECA and LSKA by 1.4% on mAP50-95, with its GFLOPs increasing by only 0.2 G, demonstrating its advantage in balancing accuracy and efficiency. Although SPPFLKC has slightly higher computational and parameter complexity than some models, it remains the leader in overall accuracy. This indicates that SPPFLKC stands out in numerous assessment measures, affirming its overall efficacy.

#### 3.4.2. Model Comparison Experiment

This study employs nine classic object detection methods: SSD, Faster R-CNN, RT-DETR, Mamba-YOLO, YOLOv5s, YOLOv7-tiny, YOLOv8n, YOLOv10n, and YOLOv11n [32,33,34]. In the object detection comparison experiments, we selected a series of lightweight object detection algorithms and compared their performance with our proposed YOLOv11-RD algorithm under identical conditions.

As shown in Table 5, under the same training conditions (dataset splitting and augmentation methods), YOLOv11n surpasses eight other networks, including YOLOv8n and YOLOv10n, in terms of precision, recall, and mAP, while achieving lower computational complexity and fewer parameters. Although YOLOv11n has slightly higher computational and parameter requirements than YOLOv7-tiny, it achieves notably better precision, recall, and mAP. Compared to the original YOLOv11n, the proposed YOLOv11-RD achieves an enhancement of 2.7% (95.8% vs. 93.3%) in mAP50 and 11.5% (76.3% vs. 68.4%) in mAP50-95, along with a 2.4% (94.6% vs. 92.4%) increase in precision. Additionally, YOLOv11-RD reduces the computational complexity by 1.1 GFLOPs (7.8 G vs. 8.9 G) and decreases the parameter count by 4.58 M (6.92 M vs. 11.5 M). Notably, YOLOv11-RD also demonstrates superior deployment efficiency on resource-constrained devices. In comparison with recent algorithms in related studies (e.g., YOLO-YSTs and RDRM-YOLO) [35,36], the proposed method shows clear superiority across all evaluation metrics.

#### 3.4.3. Ablation Experiment

In order to evaluate the performance and advantages of YOLOv11-RD, several ablation experiments were performed using various combinations of improvement modules. The evaluation criteria comprised precision, recall, mAP50, mAP50-95, the computational complexity measured in GFLOPs, and the number of parameters reported in millions. Table 6 presents the results of these ablation experiments, highlighting significant performance gains from different YOLOv11-RD enhancement modules. Moreover, by systematically dissecting diverse module combinations, we are able to undertake a comprehensive and nuanced evaluation of YOLOv11-RD’s overall performance, thereby uncovering its potential strengths and limitations.

The model featuring the SPPFLKC module demonstrated remarkable advancements of 1.8%, 1.7%, and 4.2% in the precision, mAP50, and mAP50-95 metrics, respectively, when contrasted with YOLOv11n. This highlights the profound impact of the LSKAC self-attention mechanism integrated within the SPPFLKC module on enhancing feature representation during extraction. Equally noteworthy, models incorporating the C3k2-CFCGLU and C3k2-CSCBAM showcased significant enhancements in these performance indicators while concurrently diminishing computational demands and model dimensions. In particular, relative to YOLOv11n, the model integrating both of the aforementioned modules realized a 1.6% boost in accuracy, a 1.5% rise in mAP50, and an impressive 11% elevation in mAP50-95. Furthermore, this configuration reduced the computational complexity by 1.6 GFLOPs and decreased the number of parameters by 6.32 M. The investigation reveals that the employment of the C3k2-CFCGLU and C3k2-CSCBAM not only mitigates redundancy but also significantly boosts small-object detection capabilities. Compared to YOLOv11n, the introduction of a specialized small-object detection layer resulted in a 1.4% enhancement in mAP50 and a 2.8% increment in mAP50-95, thereby substantiating the efficacy of this method in enhancing the accuracy of small-target recognition.

#### 3.4.4. Cross-Validation Experiment

In order to assess model stability, this study uses K-fold cross-validation, with the details in Table 7. During the cross-validation process, all folds shared the same hyperparameters (as listed in Table 3), and no additional tuning was performed for individual folds. The model demonstrates consistent performance across different sets through 5-fold validation. The precision values for each fold are 94.5%, 94.6%, 94.8%, 94.7%, and 94.4%, while the mAP50 values are 95.7%, 95.9%, 95.5%, 95.6%, and 95.8%. The mAP50-95 values are 76.1%, 76.3%, 76.2%, 76.5%, and 76.4%. The final average performance metrics are as follows: an average precision of 94.6%, an average mAP50 of 95.7%, and an average mAP50-95 of 76.3%. The results indicate that YOLOv11-RD not only sustains high detection accuracy across different dataset partitions but also exhibits strong generalization capabilities.

Overall, this study introduces and thoroughly evaluates the YOLOv11-RD model. The experiments indicate that the model performs outstandingly across various dataset splits, affirming its stability and generalization capabilities. Cross-validation mitigates biases from data splits and offers a more thorough evaluation of practical effectiveness.

### 3.5. Algorithm Effect Verification

This experiment selected three typical scenarios from the farmland rice disease dataset, sunny conditions, overcast weather, and dense planting, which were grouped into A, B, and C, respectively. Each group compared the detection performance of four algorithms—YOLOv11-RD, YOLOv10n, YOLOv8n, and YOLOv7-tiny—on three types of rice diseases.

From the detection result comparison, it is evident that YOLOv11-RD demonstrates significant advantages in handling complex scenarios such as sunny environments, cloudy conditions, and dense planting.

Experiments conducted in Group A under sunny conditions reveal varying performances of different algorithms in detecting small diseased areas at long distances. While YOLOv10n, YOLOv8n, and YOLOv7-tiny can detect some diseased regions, their detection results are sometimes imprecise, with lower confidence scores. In contrast, YOLOv11-RD not only reduces missed detections but also improves the recognition accuracy of tiny diseased areas, demonstrating higher reliability and precision.

In the Group B experiment under cloudy conditions, YOLOv10n, YOLOv8n, and YOLOv7-tiny exhibited a high miss rate for diseased targets and also mistakenly identified some weeds as diseases. In contrast, YOLOv11-RD demonstrated outstanding performance, accurately detecting almost all diseased targets without any false positives. This markedly improves the detection capability for small disease targets under low-light conditions, demonstrating superior reliability and accuracy.

In the Group C experiment with densely planted rice fields, YOLOv10n, YOLOv8n, and YOLOv7-tiny were able to identify disease spots with distinct leaf tip contours but struggled to detect those with indistinct boundaries or those blending into the background, especially in heavily occluded areas in the back rows. Additionally, the confidence scores of some detected frames were relatively low. In contrast, YOLOv11-RD achieved twice the detection rate for indistinct and overlapping disease spots compared to YOLOv10n, YOLOv8n, and YOLOv7-tiny and three times the detection rate for disease spots severely occluded at the base of leaves. This significantly enhances the model’s ability to detect disease spots with unclear contours, overlapping targets, and severe occlusions—especially those closely connected to other leaves with indistinct boundaries—while maintaining a high confidence level.

Table 8 summarizes the quantitative metrics (precision, recall, and mAP50-95) for each algorithm across these scenarios.

**Figure 13:**
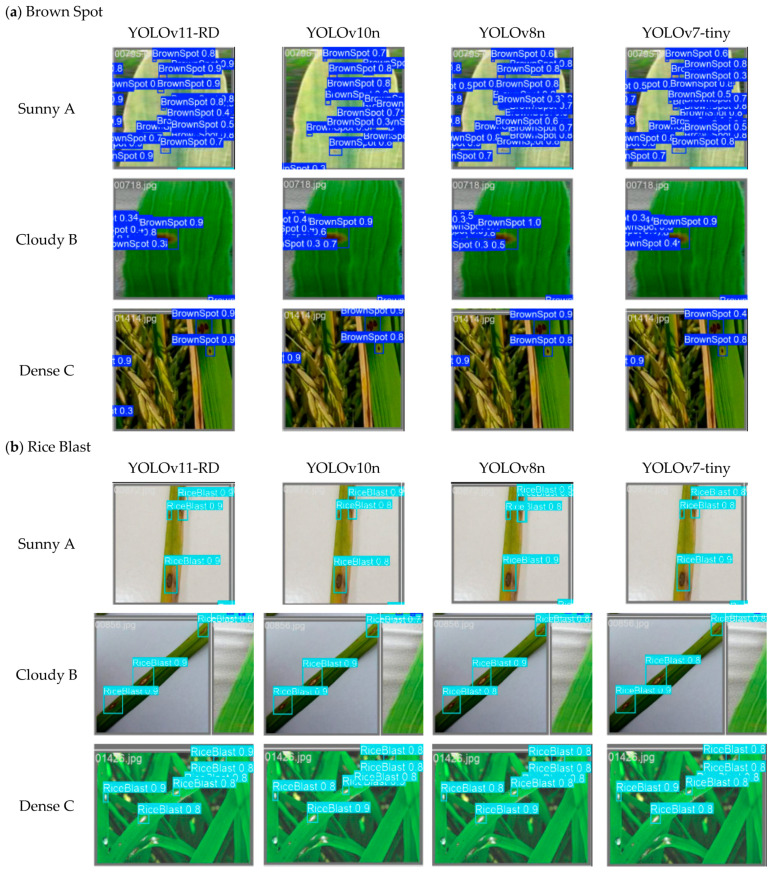

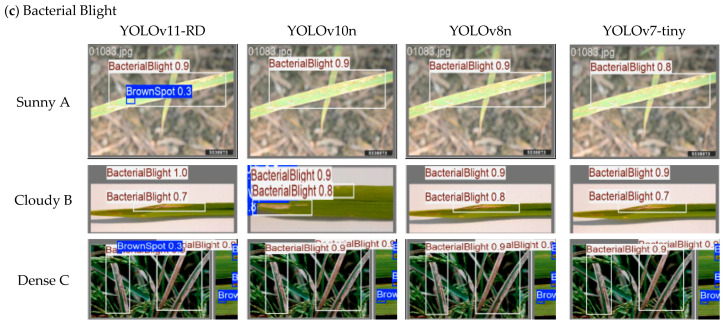
Comparison of the effect of the rice disease detection.

From the detection result comparison (Figure 13) and quantitative metrics in Table 8, YOLOv11-RD demonstrates significant advantages in handling complex scenarios. In Group A (sunny conditions), YOLOv11-RD achieves a precision of 96.2% and mAP50-95 of 78.5%, surpassing YOLOv10n by 3.5% and 7.3%, respectively. In Group B (cloudy conditions), it maintains robust performance with a recall of 93.5%, reducing false positives by 9.2% compared to YOLOv8n. For Group C (dense planting), YOLOv11-RD’s mAP50-95 (76.1%) is 8.3% higher than that of YOLOv7-tiny, highlighting its capability in detecting occluded targets.

The experimental results demonstrate that, compared to other models, YOLOv11-RD not only detects more disease spots with unclear contours or those blending into the background but also effectively manages severe occlusions. This enhancement substantially boosts the detection accuracy for small diseased areas in complex settings, providing strong support for precision agriculture.

### 3.6. Data Analysis

**Figure 14:**
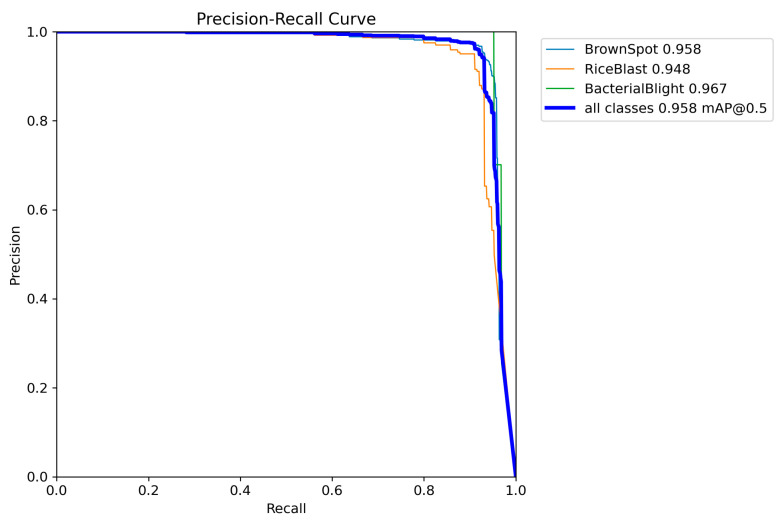
Precision-recall curve.

Figure 14 displays the precision–recall curve of YOLOv11-RD on the validation set, where precision reflects the accuracy of detections, and recall evaluates the proportion of real diseases successfully recognized. Specifically, on the validation set, the mean precision for Brown Spot, Rice Blast, and Bacterial Blight is 0.958, 0.948, and 0.967, respectively, with an overall mAP50 value of 0.958 across all categories. These results highlight that YOLOv11-RD performs exceptionally well in detecting various types of rice diseases, ensuring high accuracy and comprehensive coverage. Moreover, the model demonstrates a high degree of consistency and reliability across different disease types, particularly in recognizing disease features under complex backgrounds and at various growth stages. Further analysis of the precision–recall curve reveals that YOLOv11-RD not only excels in detecting common diseases but also shows robust capability in identifying smaller or early-stage diseases that are typically more challenging to detect under complex field conditions (e.g., occlusion, low contrast).

**Figure 15:**
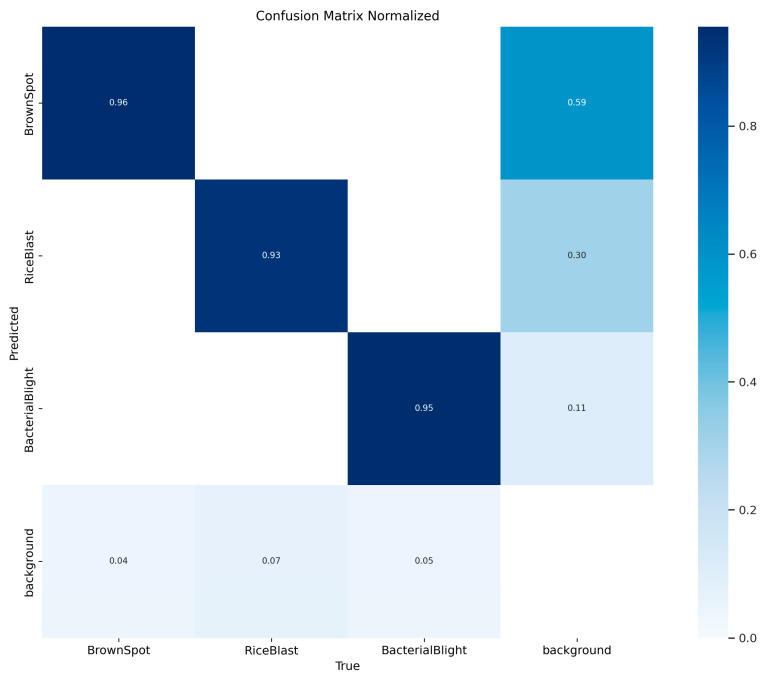
Confusion matrix.

Figure 15 presents the confusion matrix for YOLOv11-RD based on the validation set. As illustrated in the figure, the true positive (TP) rates for the three diseases are 0.96 for Brown Spot, 0.93 for Rice Blast, and 0.95 for Bacterial Blight.

It is observed that Rice Blast and Bacterial Blight exhibit cross-misclassification (estimated at about 4%), indicating the model’s limited ability to distinguish between them. This issue arises from feature similarities and data limitations. Enhancing data diversity, optimizing the attention mechanism and loss function, and applying post-processing strategies can significantly improve differentiation. Future work should prioritize fine-grained feature extraction and error case analysis for more accurate field disease diagnosis.

Through data validation, the proposed model’s performance has been confirmed, demonstrating its effectiveness in detecting small rice disease targets.

**Figure 16:**
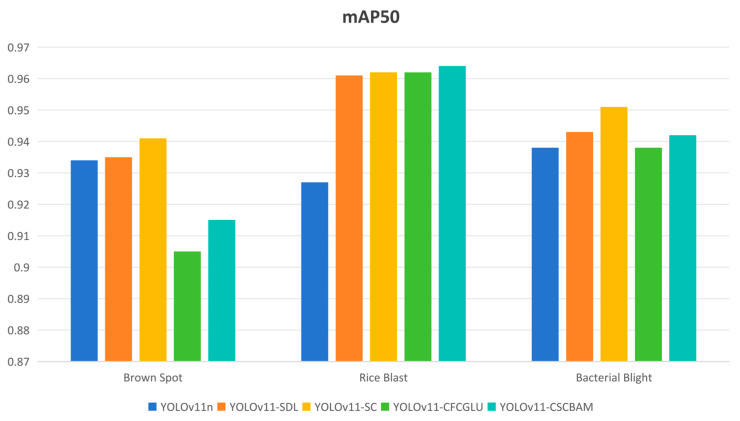
An intuitive visualization of detection accuracy among various methods.

To evaluate the recognition effectiveness of various enhancement components on different target types, Figure 16 shows the mAP for three target categories in the Rice-LeafDiseaseDetection dataset. The experimental results reveal that, compared to YOLOv11n, the detection accuracy improves after incorporating four distinct improvement strategies. Notably, the SPPFLKC attention module and small-object detection layer greatly improve the recognition accuracy of small disease targets. Even with only the C3k2-CFCGLU and C3k2-CSCBAM, the model size is reduced while still improving small-disease-target recognition. Combining these modules with the lightweight detection layer further improves and stabilizes the performance in recognizing diseases and small weed targets.

**Figure 17:**
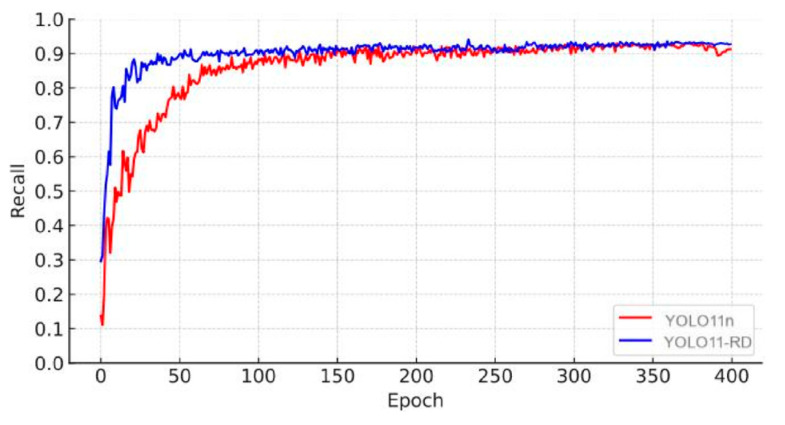
Recall trend.

**Figure 18:**
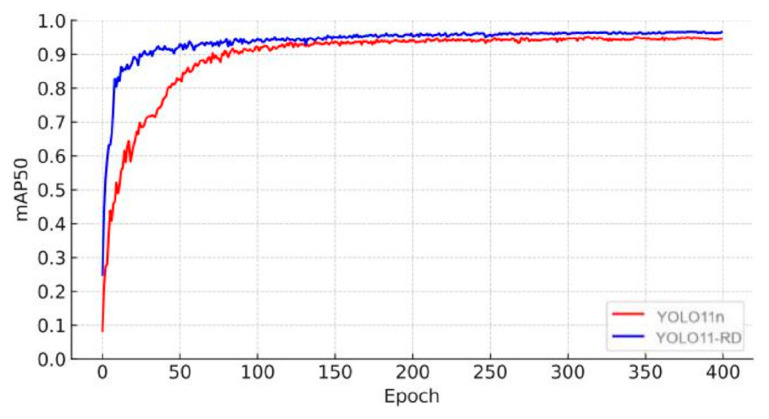
mAP50 trend.

Figure 17 and Figure 18 depict the comprehensive performance of recall and mAP50 for YOLOv11n and YOLOv11-RD after 400 training epochs. The figures show that YOLOv11-RD not only achieves more stable convergence compared to YOLOv11n but also experiences faster performance gains. Specifically, during training, YOLOv11-RD demonstrates superior learning capabilities, with precision values increasing at a notably quicker pace than YOLOv11n. This highlights YOLOv11-RD’s significant advantages in both detection accuracy and stability.

**Figure 19:**
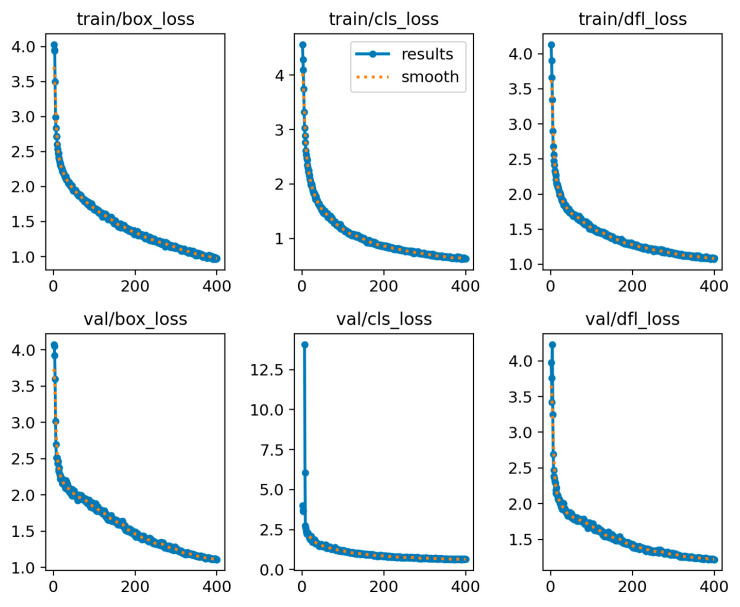
Trends in loss for the training and validation sets.

**Figure 20:**
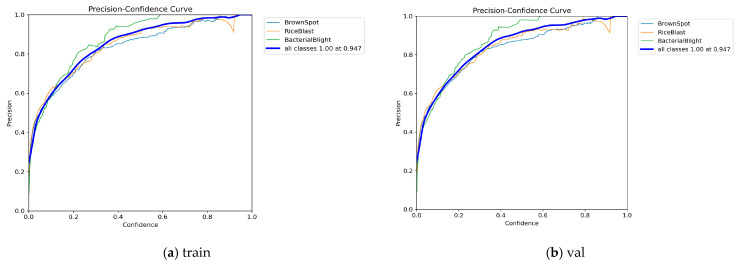
Accuracy trends for the training and validation sets.

Figure 19 and Figure 20 show the loss and accuracy curves of the YOLOv11-RD model on the training and validation sets, respectively. The loss curves indicate that both training and validation losses decrease and stabilize, demonstrating good fitting and generalization. The accuracy curves reveal that for “Brown Spot”, “Rice Blast”, and “Bacterial Blight”, the accuracy closely matches the confidence curves, with a minimal performance gap between the validation and training sets. These results highlight the model’s high consistency during training and validation, indicating no significant overfitting. Moreover, as confidence increases, the accuracy of each category improves steadily, confirming the model’s excellent generalization capabilities.

### 3.7. Performance Assessment of Training, Validation, and Test Sets

Table 9 displays the key performance indicators of the YOLOv11-RD model on the training, validation, and test sets. The experimental findings indicate that the precision and recall values demonstrate remarkable consistency across all datasets, underscoring not only the model’s efficacy in distinguishing true positives from negatives but also its exceptional adaptability to new data. Although the mAP50 scores at an IoU threshold of 0.5 are nearly identical, reflecting stable performance across various datasets, the more stringent mAP50-95 metric—which spans multiple IoU thresholds ranging from 0.5 to 0.95—still yields strikingly similar values across the three datasets. A detailed analysis of specific values across datasets shows that YOLOv11-RD demonstrates high stability and reliability in handling diverse sample distributions, especially for disease detection under complex backgrounds and fluctuating conditions. These results confirm the model’s excellent performance across metrics while highlighting its broad applicability and robustness in real-world scenarios.

**Figure 21:**
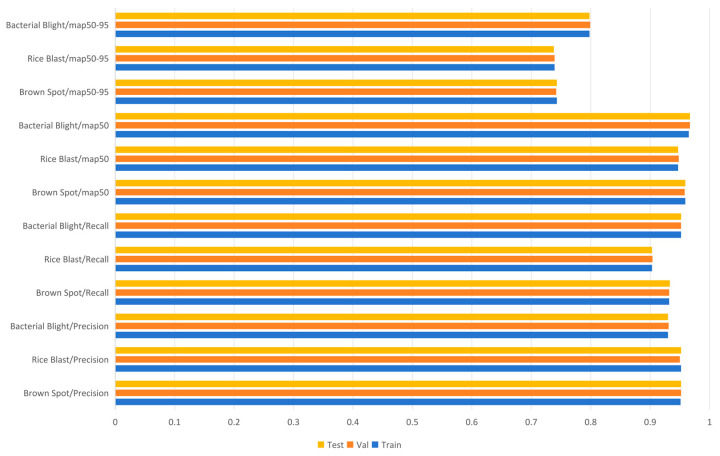
Performance metrics for each class in training, validation, and test sets.

Figure 21 shows the detection results of YOLOv11-RD for three target categories across three sets. Metrics like precision, recall, mAP50, and mAP50-95 are highly consistent across datasets, indicating stable and accurate performance. YOLOv11-RD demonstrates excellent generalization without overfitting, delivering reliable results in complex backgrounds and diverse sample distributions. Core indicators highlight its superior capabilities, making it ideal for object detection in intricate environments.

### 3.8. Practical Deployment Validation

To validate the impact of computational complexity reduction on real-world deployment, experiments were conducted on embedded devices commonly used in agricultural settings, including the NVIDIA Jetson Nano (4 GB RAM) and Raspberry Pi 4B (4 GB RAM). Both the original YOLOv11n and the improved YOLOv11-RD models were deployed using TensorRT optimization, and their performance was evaluated under real-time field conditions. On the Jetson Nano, YOLOv11-RD achieved 23.5 FPS, outperforming YOLOv11n’s 18.2 FPS, demonstrating a 29% improvement in real-time processing capability. YOLOv11-RD reduced the memory usage by 32% (from 1.8 GB to 1.2 GB), significantly alleviating operational pressure on memory-constrained devices. Measured via a power monitor, YOLOv11-RD consumed an average of 3.2 W, 18% lower than YOLOv11n’s 3.9 W, thereby substantially extending the battery life for drones.

YOLOv11-RD was integrated into a DJI Agras T30 drone for real-time rice disease monitoring. The model successfully detected diseases (e.g., Rice Blast) in 320 × 320 resolution images at 20 FPS, with latency below 50 ms. Compared to YOLOv11n, the lightweight design reduced data transmission overhead by 22%, ensuring stable performance even under 4 G network bandwidth constraints.

## 4. Conclusions

Practically, YOLOv11-RD effectively addresses the limitations of insufficient feature expression and low precision in traditional rice disease detection by introducing innovative modules such as SPPFLKC and C3k2-CFCGLU, significantly enhancing detection capabilities for small targets and dense areas in complex farmland environments. From a societal perspective, this model enables highly accurate real-time detection, reducing diagnostic delays, preventing disease spread, optimizing labor allocation, and cutting costs. The experimental results show improvements of 2.7% and 11.5% in mAP50 and mAP50-95, respectively, with a reduction of 4.58 M in the parameters and 1.1 G in the computational cost. This lightweight design not only improves accuracy but also ensures efficient deployment on resource-constrained systems. Future research should focus on optimizing the model’s generalization ability, deeper integration with edge computing devices, and exploring multi-modal data (e.g., spectral images, meteorological data) to further enhance detection performance, driving rice disease detection technology toward greater intelligence and efficiency.

## Figures and Tables

**Table 1:** Raw data count for each category.

Category	Number
Rice Blast	2353
Brown Spot	1215
Bacterial Blight	1432

**Table 2:** Statistics of sample counts per category after data augmentation.

Category	Number
Brown Spot	1600
Rice Blast	1200
Bacterial Blight	1200

**Table 3:** Parameter setup.

Parameters	Settings	Parameters	Settings
Optimizer	SGD	lrf	0.01
Epochs	300	weight_decay	0.005
Batchsize	32	momentum	0.937
Workers	4	warmup_epochs	3
Imgsz	640	warmup_momentum	0.8
lr0	0.01	close_mosaic	0

**Table 4:** Comparison of attention mechanisms.

Model	Precision (%)	Recall (%)	map50 (%)	mAP 50-95 (%)	GFLOPs	Params /M
SE	94.1	91.2	94.8	73.3	8.7	12.13
ELA	93.7	90.3	93.4	72.9	8.5	10.28
ECA	93.6	90.1	92.9	73.4	8.4	10.02
EMA	93.4	89.9	92.5	72.8	8.5	10.45
LSKA	93.8	90.5	93.7	72.5	8.8	13.06
SimAM	93.9	90.8	94.3	73.6	8.7	12.25
SPPFLKC	94.4	91.6	95.1	74.8	8.6	11.05

**Table 5:** Model comparison experiment.

Model	Precision (%)	Recall (%)	mAP50 (%)	mAP50-95 (%)	GFLOPs	Params /M
SSD	79.2	26.3	45.9	57.1	36.8	47.2
Faster R-CNN	83.7	72.4	75.8	63.6	205.2	105
RT-DETR	91.5	91.3	92.1	72.4	9.6	11.8
Mamba-YOLO	91.9	90.7	91.3	71.5	10.5	12.4
YOLOv5s	80.3	76.2	79.6	58.2	17.4	28.1
YOLOv7-tiny	81.6	78.3	82.3	64.3	6.6	6.8
YOLOv8n	90.8	88.6	90.8	70.8	9.3	15.8
YOLOv10n	91.3	90.5	92.5	72.6	9.2	12.3
YOLOv11n	92.4	88.5	93.3	68.4	8.9	11.5
YOLO-YSTs	83.2	83.2	86.8	41.3	8.8	3.02
RDRM-YOLO	94.3	89.6	93.5	70.5	10.2	7.9
YOLOv11-RD	94.6	93.1	95.8	76.3	7.8	6.92

**Table 6:** Ablation experiments.

SDL	SPPFLKC	C3k2-CFCGLU	C3k2-CSCBAM	Precision (%)	Recall (%)	mAP50 (%)	mAP 50-95 (%)	GFLOPs	Params /M
×	×	×	×	92.4	88.5	93.3	68.4	8.9	11.5
√	×	×	×	93.7	90.5	94.6	70.3	9.1	12.14
×	√	×	×	94.1	91.8	94.9	71.3	9.1	12.05
×	×	√	×	93.7	91.2	94.6	73.9	8.3	7.73
×	×	×	√	93.6	90.4	94.5	74.7	8.2	7.61
√	√	×	×	94.2	90.8	94.9	74.8	9.4	13.39
√	×	√	×	93.4	91.3	95.1	73.3	8.4	8.73
√	×	×	√	94.3	91.5	95.2	73.2	8.3	8.48
×	√	√	×	94.2	90.8	94.6	74.1	8.3	8.53
×	√	×	√	94.1	91.5	95.4	74.4	8.4	8.61
×	×	√	√	93.9	91.4	94.7	75.9	7.3	5.18
√	√	√	×	93.8	91.8	95.2	75.3	8.7	9.39
√	√	×	√	93.8	92.3	95.3	75.2	8.6	9.12
√	×	√	√	94.3	92.5	95.3	76.1	7.6	5.56
×	√	√	√	94.5	92.9	95.5	75.5	7.7	5.72
√	√	√	√	94.6	93.1	95.8	76.3	7.8	6.92

Note: “×” indicates a component’s absence, while “✓” shows its presence. SDL is the Small Detection Layer.

**Table 7:** Outcomes of the cross-validation.

Folds	Precision (%)	Recall (%)	mAP50 (%)	mAP50-95 (%)
1	94.5	93.1	95.7	76.1
2	94.6	93.0	95.9	76.3
3	94.8	93.2	95.5	76.2
4	94.7	93.3	95.6	76.5
5	94.4	92.9	95.8	76.4
Average	94.6	93.1	95.7	76.3

**Table 8:** Quantitative performance comparison of algorithms in Groups A, B, and C.

Group	Model	Precision (%)	Recall (%)	mAP50-95 (%)
A (sunny)	YOLOv11-RD	96.2	94.8	78.5
	YOLOv10n	92.7	89.3	71.2
	YOLOv8n	90.5	87.6	69.8
	YOLOv7-tiny	85.4	82.1	63.4
B (cloudy)	YOLOv11-RD	95.8	93.5	77.9
	YOLOv10n	91.4	88.7	70.5
	YOLOv8n	89.2	85.9	68.1
	YOLOv7-tiny	83.6	80.3	61.2
C (dense)	YOLOv11-RD	94.3	92.1	76.1
	YOLOv10n	89.8	86.4	67.8
	YOLOv8n	87.5	84.2	65.3
	YOLOv7-tiny	81.9	78.0	59.7

**Table 9:** Performance metrics for the partition set in its entirety.

Set	Precision (%)	Recall (%)	mAP50 (%)	mAP50-95 (%)
Train	95.2	92.8	95.7	75.8
Val	95.1	93.1	95.8	76.3
Test	95.1	93.3	95.8	75.9

## Data Availability

The datasets used and analyzed during the current study are available from the corresponding author upon reasonable request. We have made the dataset public on the following website: https://github.com/TengHX99/YOLOv11-RD-data (accessed on 25 March 2025).

## Abbreviations

The following abbreviations are used in this manuscript:

RD	Rice disease
YOLOv11	You Only Look Once version 11
SPPFLKC	Modified SPPF module with Large-Separable-Kernel Attention with Convolution
C3k2-CFCGLU	Modified C3k2 block with CAFormer and CGLU
CAFormer	Convolutional Attention Former
CGLU	Convolutional Gated Linear Unit
C3k2-CSCBAM	Modified C3k2 block with CSCBAM
CSCBAM	Channel–Spatial Combined Attention Module
C3k2	Cross Stage Partial Bottleneck with 3 Conv layers and Key-point Detection
LZKAC	Large-Kernel Attention with Convolution
SPPF	Spatial Pyramid Pooling Fast
LKA	Large-Kernel Attention
LSKA	Large-Separable-Kernel Attention
MaxPool2d	Max pooling 2D
AvgPool2d	Average pooling 2D
SCConv	Split Convolution
GN	Group Normalization
DW	Deep Width
GWConv	Group Convolution
PWConv	Point-by-point Convolution
MLP	Multi-layer perceptron
BN	Batch Normalization
ReLU	Rectified Linear Unit
mAP	Mean average precision

## References

[1] Erenstein O., Jaleta M., Mottaleb K.A., Sonder K., Donovan J., Braun H.-J. (2022). Global trends in wheat production, consumption and trade. Wheat Improvement: Food Security in a Changing Climate.

[2] Wei T., Xu L.J.S.D. (2025). China food security comprehensive assessment dataset 2012–2022. Sci. Data.

[3] Rudiyanto, Minasny B., Shah R.M., Che Soh N., Arif C., Indra Setiawan B. (2019). Automated near-real-time mapping and monitoring of rice extent, cropping patterns, and growth stages in Southeast Asia using Sentinel-1 time series on a Google Earth Engine platform. Remote Sens..

[4] Xu S., Zhu X., Chen J., Zhu X., Duan M., Qiu B., Wan L., Tan X., Xu Y.N., Cao R. (2023). A robust index to extract paddy fields in cloudy regions from SAR time series. Remote Sens. Environ..

[5] Fahad S., Adnan M., Noor M., Arif M., Alam M., Khan I.A., Ullah H., Wahid F., Mian I.A., Jamal Y. (2019). Advances in rice research for abiotic stress tolerance. Major Constraints for Global Rice Production.

[6] Qi G., Zhang Y., Wang K., Mazur N., Liu Y., Malaviya D. (2022). Small object detection method based on adaptive spatial parallel convolution and fast multi-scale fusion. Remote Sens..

[7] Bansal M., Kumar M., Kumar M., Kumar K. (2021). An efficient technique for object recognition using Shi-Tomasi corner detection algorithm. Soft Comput..

[8] Wang G., Zhuang Y., Chen H., Liu X., Zhang T., Li L., Dong S., Sang Q. (2021). FSoD-Net: Full-scale object detection from optical remote sensing imagery. IEEE Trans. Geosci. Remote Sens..

[9] Vijayakumar A., Vairavasundaram S. (2024). Yolo-based object detection models: A review and its applications. Multimed. Tools Appl..

[10] Diwan T., Anirudh G., Tembhurne J.V. (2023). Object detection using YOLO: Challenges, architectural successors, datasets and applications. Multimed. Tools Appl..

[11] Wang C.-Y., Bochkovskiy A., Liao H.-Y.M. YOLOv7: Trainable bag-of-freebies sets new state-of-the-art for real-time object detectors. Proceedings of the IEEE/CVF Conference on Computer Vision and Pattern Recognition.

[12] Liu W., Anguelov D., Erhan D., Szegedy C., Reed S., Fu C.-Y., Berg A.C. Ssd: Single shot multibox detector. Proceedings of the Computer Vision–ECCV 2016: 14th European Conference.

[13] Girshick R. Fast r-cnn. Proceedings of the IEEE International Conference on Computer Vision.

[14] Ren S., He K., Girshick R., Sun J. (2016). Faster R-CNN: Towards real-time object detection with region proposal networks. IEEE Trans. Pattern Anal. Mach. Intell..

[15] Cai Z., Vasconcelos N. Cascade r-cnn: Delving into high quality object detection. Proceedings of the IEEE Conference on Computer Vision and Pattern Recognition.

[16] Xue Z., Xu R., Bai D., Lin H. (2023). YOLO-tea: A tea disease detection model improved by YOLOv5. Forests.

[17] Jia L., Wang T., Chen Y., Zang Y., Li X., Shi H., Gao L. (2023). MobileNet-CA-YOLO: An improved YOLOv7 based on the MobileNetV3 and attention mechanism for Rice pests and diseases detection. Agriculture.

[18] Sangaiah A.K., Yu F.-N., Lin Y.-B., Shen W.-C., Sharma A. (2024). UAV T-YOLO-rice: An enhanced tiny YOLO networks for rice leaves diseases detection in paddy agronomy. IEEE Trans. Netw. Sci. Eng..

[19] Li H., Wu A., Jiang Z., Liu F., Luo M. Improving object detection in YOLOv8n with the C2f-f module and multi-scale fusion reconstruction. Proceedings of the 2024 IEEE 6th Advanced Information Management, Communicates, Electronic and Automation Control Conference (IMCEC).

[20] Lau K.W., Po L.-M., Rehman Y.A.U. (2024). Large separable kernel attention: Rethinking the large kernel attention design in cnn. Expert Syst. Appl..

[21] Guo M.H., Lu C.Z., Liu Z.N., Cheng M.M., Hu S.M. (2023). Visual attention network. Comput. Vis. Media.

[22] Li J., Wen Y., He L. Scconv: Spatial and channel reconstruction convolution for feature redundancy. Proceedings of the IEEE/CVF Conference on Computer Vision and Pattern Recognition.

[23] Dai Y., Li C., Su X., Liu H., Li J. (2023). Multi-scale depthwise separable convolution for semantic segmentation in street–road scenes. Remote Sens..

[24] Zhang A., Jia L., Wang J., Wang C. (2023). Sar image classification using gated channel attention based convolutional neural network. Remote Sens..

[25] Dong J., Zhuang D., Huang Y., Fu J. (2009). Advances in multi-sensor data fusion: Algorithms and applications. Sensors.

[26] Lv H., Chen J., Pan T., Zhang T., Feng Y., Liu S. (2022). Attention mechanism in intelligent fault diagnosis of machinery: A review of technique and application. Measurement.

[27] Ma R., Chen J., Feng Y., Zhou Z., Xie J. (2025). ELA-YOLO: An efficient method with linear attention for steel surface defect detection during manufacturing. Adv. Eng. Inform..

[28] Ni H., Shi Z., Karungaru S., Lv S., Li X., Wang X., Zhang J. (2023). Classification of typical pests and diseases of Rice based on the ECA attention mechanism. Agriculture.

[29] Hao W., Ren C., Han M., Zhang L., Li F., Liu Z. (2023). Cattle body detection based on YOLOv5-EMA for precision livestock farming. Animals.

[30] Wang C., Wang Y. (2024). Sensors. SLGA-YOLO: A Lightweight Castings Surface Defect Detection Method Based on Fusion-Enhanced Attention Mechanism and Self-Architecture. Sensors.

[31] Mahaadevan V., Narayanamoorthi R., Gono R., Moldrik P. (2023). Automatic identifier of socket for electrical vehicles using SWIN-transformer and SimAM attention mechanism-based EVS YOLO. IEEE Access.

[32] Zhai S., Shang D., Wang S., Dong S. (2020). DF-SSD: An improved SSD object detection algorithm based on DenseNet and feature fusion. IEEE Access.

[33] Wang S., Jiang H., Li Z., Yang J., Ma X., Chen J., Tang X. (2024). Phsi-rtdetr: A lightweight infrared small target detection algorithm based on UAV aerial photography. Drones.

[34] Qi K., Yang Z., Fan Y., Song H., Liang Z., Wang S., Wang F. (2025). Detection and classification of Shiitake mushroom fruiting bodies based on Mamba YOLO. Sci. Rep..

[35] Huang Y., Liu Z., Zhao H., Tang C., Liu B., Li Z., Wan F., Qian W., Qiao X. (2025). YOLO-YSTs: An Improved YOLOv10n-Based Method for Real-Time Field Pest Detection. Agronomy.

[36] Li P., Zhou J., Sun H., Zeng J. (2025). RDRM-YOLO: A High-Accuracy and Lightweight Rice Disease Detection Model for Complex Field Environments Based on Improved YOLOv5. Agriculture.

